# Self-Processing and Self-Face Reaction Time Latencies: A Review

**DOI:** 10.3390/brainsci11111409

**Published:** 2021-10-26

**Authors:** Gordon G. Gallup, Steven M. Platek

**Affiliations:** 1 Department of Psychology, State University of New York at Albany, Albany, NY 12222, USA; 2 Psychology, Georgia Gwinnett College, Lawrenceville, GA 30024, USA; splatek@gmail.com

**Keywords:** self-face reaction time latencies, cross-modal self-recognition, schizophrenia as a self-processing disorder

## Abstract

In this article, we detail the advantages of self-face identification latencies over more traditional tests of mirror self-recognition. Using reaction time latencies (measured in milliseconds) to identify different dimensions of the self, instead of relying on a simple dichotomous pass/fail mirror mark-test outcome, enables investigators to examine individual differences in self-processing time. This is a significant methodological step forward with important implications. The point of departure for our article is to detail research we and others have conducted on latencies for self-face identification, to show how self-processing occurs in the right side of the brain, how schizophrenia is a self-processing disorder, how self-face reaction time latencies implicate the existence of an underlying multiple modal self-processing system, and to explore ideas for future research.

## 1. Information about the Self Is Located on the Right Side of the Brain

**Figure 1:**
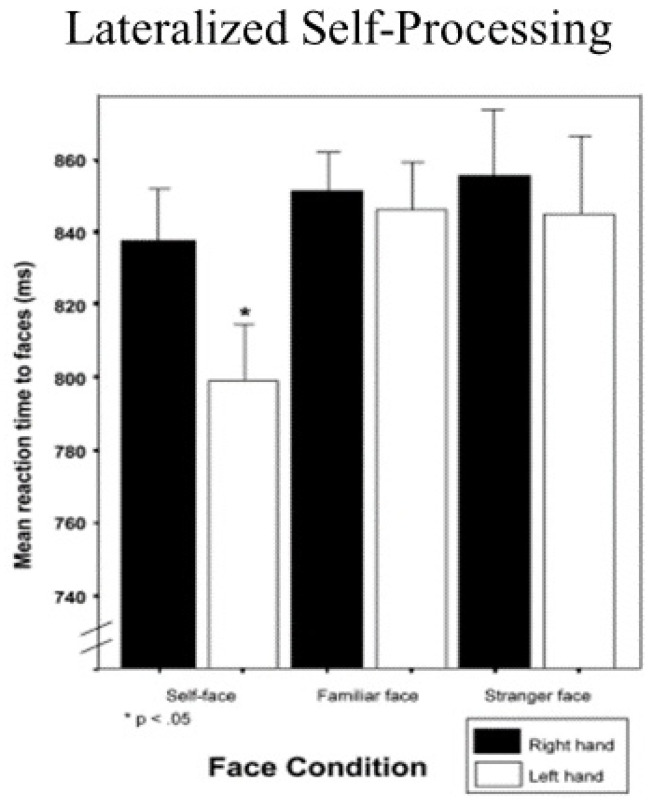
Left-hand advantage in reaction times to self-face recognition.

Some time ago, Keenan, McCutcheon, Freund, Gallup, Sanders, and Pascual-Leone [1] pioneered the development of a unique self-face reaction time paradigm. In their study, men and women were individually confronted with facial photographs of different people on a computer screen. The subject’s task was to press a particular key on the keyboard as fast as they could to indicate whether it was their face, a face of a friend, a stranger, or someone who was famous. When right-handed subjects were instructed to respond with their right hand, no differences in reaction times to self or other faces were found. However, when they were told to respond with their left hand, reaction time latencies to their own faces were faster than to other faces (see Figure 1, which is drawn from [2]).

Because of hemispheric contra-lateral control, where the right side of the brain tends to control movement on the left side of the body and the left side of the brain controls movement on the right side of the body, these results were interpreted to indicate a right-hemisphere bias for self-face processing. The rationale for this is as follows. It takes more time in milliseconds to respond to information about the self with the right hand because that information about the self must be sent across the corpus callosum to the left hemisphere before it can trigger a response to the keyboard with the right hand. Conversely, information about the self is in the same side of the brain that controls the left hand and therefore triggers a response significantly faster. In an absolute sense, these time differences are small but consistent, and important when considering the speed of neural transmission. Interestingly, this effect might be tempered by age. Zhang and Zhou [3] showed that the self-face reaction time advantage was lessened with older self-face images. The authors suggest that this may be related to a relatively less positive impression of oneself as one ages. Bortolon and Raffard [4] did a meta-analysis and summarize much of the self-face reaction time literature that is not covered in our paper.

In contrast to traditional measures of mirror self-recognition, which is typically assessed in an all or none/pass–fail manner, one of many ways to illustrate how self-face identification latencies can be used to measure an underlying quantitative continuum of individual differences in self-awareness is to examine the localization of self-recognition in the brains of neuropsychiatric patient populations, such as patients with schizophrenia.

Although many people tend to simplify and think about the world in a dichotomous all or none, good or bad, right or wrong fashion, this pass/fail mentality does not do justice to a complex world, and schizophrenia is no exception. Just as normality and abnormality are represented by underlying differences in degree rather than kind, the same applies to schizophrenia. This is why many psychiatrists and clinical psychologists now agree that schizophrenia is on a spectrum. The schizotypal personality questionnaire (SPQ) consists of 76 true/false questions about the self, such as “When you see people talking to each other, do you often wonder if they are talking about you?” It is important to acknowledge that the answer to some of these questions can be adaptive in certain contexts. For example, “Do you ever get nervous when someone is walking behind you?” However, the answer to any particular question is not definitive. Everyone answers some of these questions in the affirmative. It is the person’s overall score based on how many of these questions are answered as being true that is important. These scores can be used to identify a continuum of individual differences in premorbid schizophrenic-like thinking. As evidence that scores on the SPQ have a genetic component, unaffected relatives of schizophrenic patients score higher on the SPQ than family members of non-schizophrenic patients [5].

## 2. Schizophrenia as a Self-Processing Disorder

There is growing evidence that people with schizophrenia often cannot identify the source of their own behavior. If a person with schizophrenia is shown their own hand in real time on a computer monitor that is positioned next to another person’s hand and asked to identify which belongs to them, they cannot distinguish their hand from the other person’s hand on the screen. If asked to move their hand as they watch the images of the two hands on the monitor, they are still unable to determine which hand is theirs [6]. In other words, they appear to be incapable of identifying the source of their own behavior. 

This can be illustrated in a variety of other ways as well. Auditory hallucinations, which often take the form of hearing voices, are one of the diagnostic cornerstones of schizophrenia. There is growing evidence that just like most people, schizophrenics carry on covert conversations with themselves. Because schizophrenics are oblivious to the source of their own behavior, however, they often attribute these conversations/voices to other people [7].

Another compelling example of the same effect involves self-tickling. Many people experience being tickled when they are tickled by others, but not when they tickle themselves. However, schizophrenics and people with high scores on the SPQ act as if they are being tickled by someone else when instructed to tickle themselves [7,8,9]. This is another obvious instance of an inability to identify the source of one’s own behavior.

Although it is not meant to be demeaning, it is theoretically relevant to point out that, just like schizophrenics, rhesus monkeys fail to recognize themselves in mirror (see [8,10]) and they also act as if they cannot identify the source of their own behavior. In an experiment with both chimpanzees and rhesus monkeys that had received extensive prior experience with mirrors, both groups were trained to find food on the other side of an opaque barrier by inserting their arm through an opening with the use of a mirror positioned on the other side of the barrier to guide their hand to food located on platforms attached to the other side of the barrier that could not otherwise be seen. Chimpanzees easily solved the problem and used the mirror to find and access the food. In contrast, the rhesus monkeys failed to solve the problem, and when they saw the reflection of their hand in the mirror approach the hidden food they sometimes vocalized and threated the reflection of their hand as if it were the hand of another monkey [8]. These findings are clearly reminiscent of the research with schizophrenics which shows that by moving their hand they still cannot distinguish their own hand from the hand of someone else in a mirror.

By using sodium amobarbital to temporarily deactivate parts of the cortex, it has been shown that when the left hemisphere is anesthetized, people have no trouble identifying their hand on a computer monitor. When the right hemisphere is deactivated, however, they act just like rhesus monkeys, and not only fail to recognize their own hand, but attribute the hand they see on the monitor to a stranger [11]. In still another dramatic study using sodium amobarbital, an experiment was performed to localize self-face processing in the brain. Using epileptic patients who were being evaluated for possible brain surgery, Julian Keenan and his colleagues morphed each of the patient’s faces with a famous person (Marilyn Monroe for women, and Bill Clinton for men). The patients were then individually shown their face combined in a 50% morph with the famous person’s face and asked to identify which face they were seeing when one or the other cortical hemispheres was deactivated. When the left hemisphere was anesthetized, all five of the patients indicated that they were seeing their own face, but when the right hemisphere was deactivated, four out of five said that they saw the face of either Marilyn Monroe or Bill Clinton

**Figure 2:**
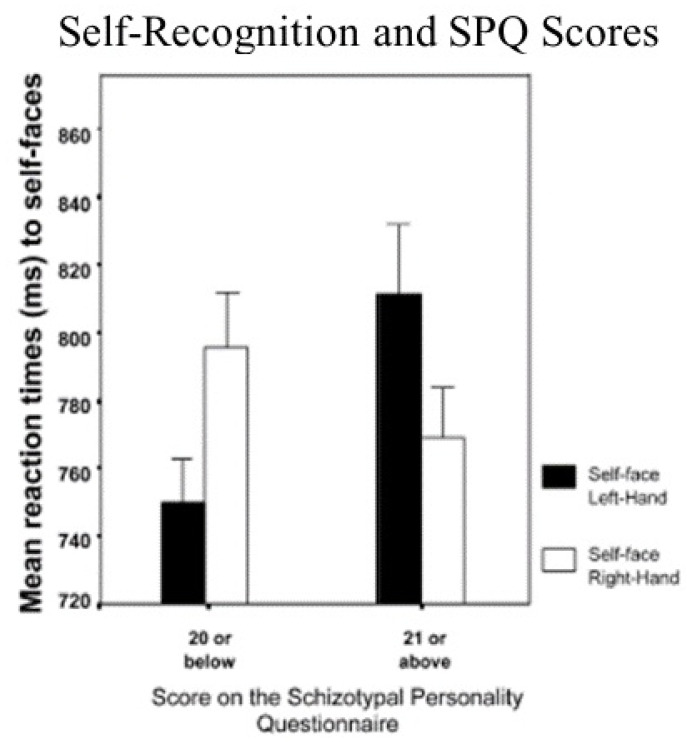
Effect of SPQ scores on self-face recognition reaction times.

To determine if the premorbid schizophrenic-like symptoms, as measured by schizotypal personality traits, might be lateralized in the brain, Platek and Gallup [12] administered the SPQ to a mixed-sex, random sample of right-handed undergraduates who were also tested using the self-face reaction time latency paradigm. Consistent with the Keenan et al. [1] study, those with low SPQ scores (i.e., low on schizophrenic-like symptomatology) showed shorter self-face identification latencies when they responded with their left hand as compared to their right hand. In contrast, students with high SPQ scores showed just the opposite effect, and it took them longer to identify their own faces when they responded with their left hand (see Figure 2). Indeed, there was a positive correlation of 0.408 between the SPQ scores and how long it took students to identify their own faces with their left hand. These results suggest that people who tend to think more like schizophrenics show impaired, rather than enhanced self-processing on the right side of the brain. Unlike mirror self-recognition, these results also constitute a compelling illustration of how underlying individual differences in self-processing exist on a continuum (see Figure 2).

In a follow-up study, Platek, Myers, Critton, and Gallup [13] examined the length of time in milliseconds it took for people to make decisions about whether a variety of different personality traits (such as intelligent, dishonest, ambitious, dependable, shy) applied to themselves (Table 1). The paradigm was one in which another sample of right-handed male and female college students were asked to fill out the SPQ, and were then shown printed individual personality characteristics presented on a computer screen and instructed to rapidly press one key on the keyboard if it applied to them, or a different key if it did not.

**Figure 3:**
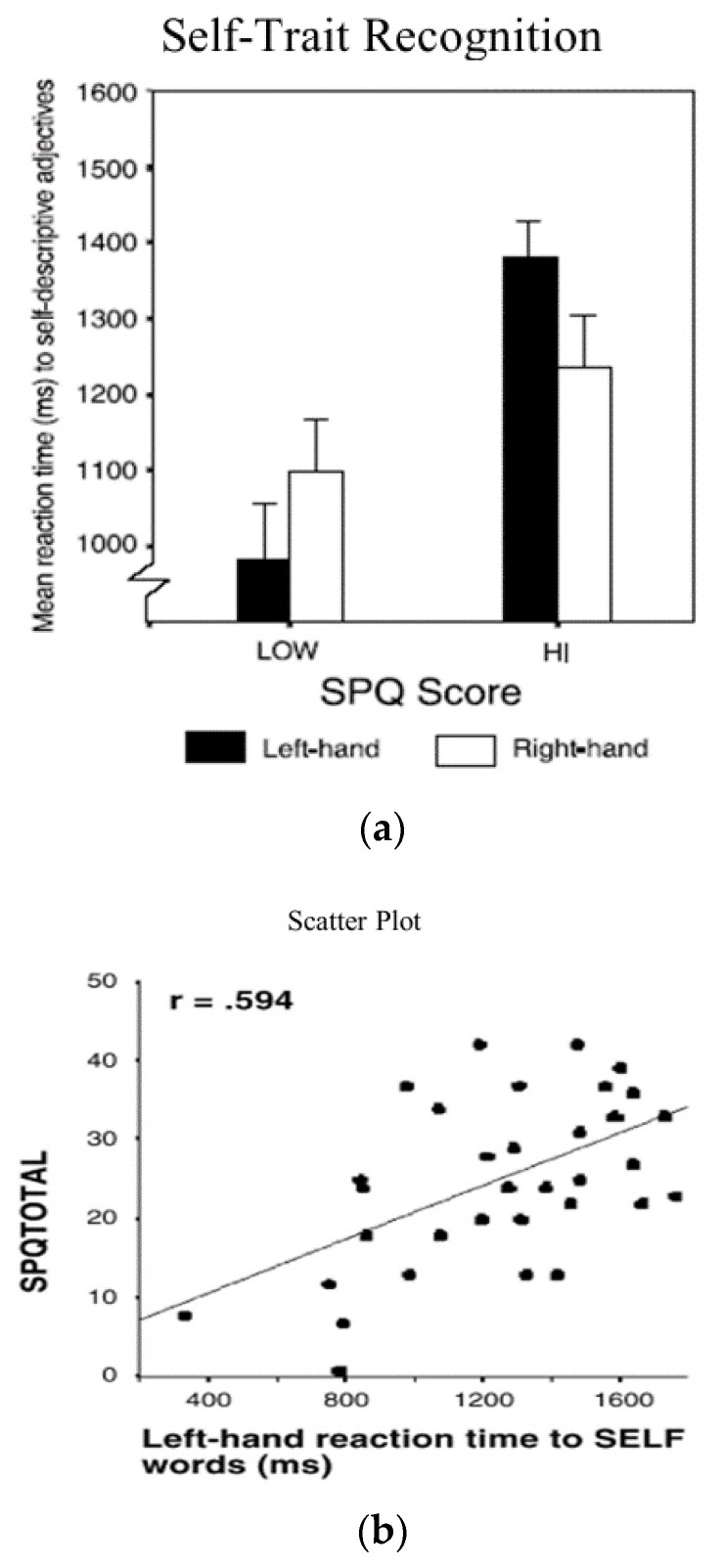
(**a**) Effects of SPQ scores on reaction times to self-trait words: (**b**) correlation between SPQ scores and left-hand reaction times to self-trait descriptors.

The results are depicted in Figure 3b,c. The results for self-trait identification latencies among students with low SPQ scores replicates the results we found with self-face identification in showing that there was a pronounced left-hand (right-hemisphere) advantage to picking self-trait adjectives. The same parallel finding was true for participants with high SPQ scores, where there was a pronounced disadvantage for self-trait identification with the left hand. Across the entire sample, there was a very substantial positive correlation of +0.594 between SPQ scores and self-trait reaction time latencies for students who responded with their left hand. By comparing the results shown in Figure 1 with Figure 3b, notice also that the latencies for self-trait identification across all conditions were 200 or more milliseconds longer than was true for self-face identification.

These results clearly extend the presence of individual differences in lateralized self-processing to another domain. As evidenced by much longer self-trait identification latencies, the ability to distinguish between appropriate and inappropriate self-descriptive adjectives goes well beyond mere self-face identification. Self-trait identification involves distinguishing between far more complex aspects of the self and further reinforces the conclusion that (1) self-processing is localized in the right hemisphere, and (2) that there is a right-hemisphere self-processing impairment/deficit in students with premorbid schizophrenic-like traits. 

In a follow-up study, we were able to show that individuals who scored highly on the SPQ were also worse at answering questions about mental state attribution. This effect was also highly correlated with the susceptibility to contagious yawning. Those individuals that had faster left-hand reaction times to self-face identification were more likely to show contagious yawning. Similarly, individuals with faster left-hand reaction times to self-face identification were more likely to answer correctly on faux pas-type theory of mind questions. What is particularly interesting is that individuals that scored higher on the SPQ were slower at self-face reaction times and less likely to show contagious yawning (Table 2). 

It is interesting to note that the paper we published (Platek and Gallup [12]) on self-face reaction time latencies, which showed that normal subjects had a left-hand self-face reaction time advantage and that this phenomenon was reversed for subjects with premorbid schizophrenic-like tendencies (i.e., had high scores on the SPQ), generated considerable interest among researchers interested in schizophrenia and inspired several other articles. The majority of these articles, eight in addition to our own (Platek and Gallup [12], Platek et al. [13], Platek et al. [14], Bortolon et al. [15], Jia et al. [16], Garcia-Alverez et al. [17], Klein et al. [18], Heinishch et al. [19], Pauly et al. [20]), report results that were consistent with our findings, while five papers (Veluw et al. [21], Kochs et al. [22], Bortolon et al. [23], Bortolon et al. [24], Zhang et al. [25]) either failed to find an effect or obtained results that were contrary to our findings.

## 3. Self-Face and Mental State Attribution in Patients with Schizophrenia and Their Family Members

Irani et al. [26] measured self-face reaction times and responses to the mind in the eyes test [27], which is a measure of the ability to infer the emotional state of people based on their facial expression, and has been widely used as a measure of mental state attribution. They found that when it came to self-face recognition, people clinically diagnosed with schizophrenia were slowest at self-face identification. In fact, in the case of family members, the results showed that unrelated controls were faster than family members of affected individuals who in turn were faster than schizophrenic patients. The same held true for responses on the mind in the eyes test. This bolsters our argument that latencies to response to self-face and mental state attribution information have a genetic component and exist on a neurocognitive continuum. 

## 4. Smelling Yourself

Platek, Burch, and Gallup [28] found that women were better at identifying their own body odor than men. They also demonstrated that affective responses to self- versus non-self-odors were modulated by this effect. It is interesting to speculate about whether olfactory self-recognition would be negatively affected in female participants that score highly on schizotypal personality traits. It is well documented that olfactory deficits are common among people who are diagnosed with schizophrenia, but not well understood [29].

The similarities between patients with frontal lobe damage and schizophrenia further bolsters the claim that schizophrenia is likely to represent a condition with a particularly robust self-processing deficit (see Table 3). 

## 5. The Multi-Modal Nature of Self-Processing Information

The way the brain represents the self is not simply a visual representation of a person’s face, but a more holistic representation of various aspects of oneself. This is evident in one’s ability to recognize their own name in a crowded room where everyone is talking. This “cocktail party effect” highlights the importance of one’s own name. A person’s name is so psychologically salient that hearing one’s own name in a crowded room cuts through the background noise and hijacks one’s neural attentional system. 

**Figure 4:**
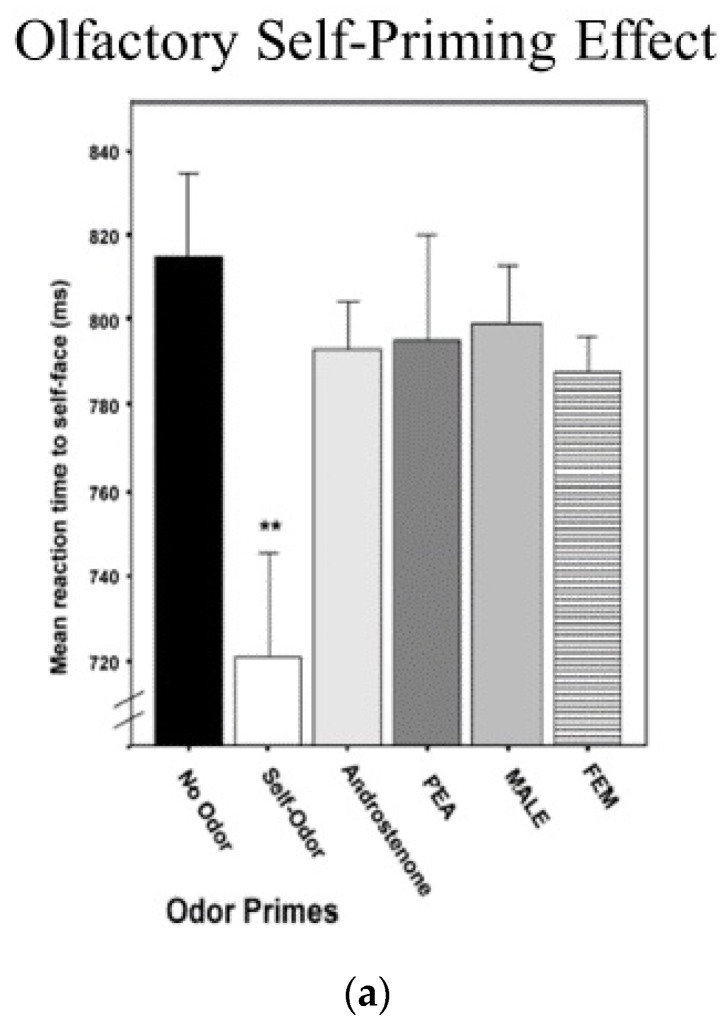

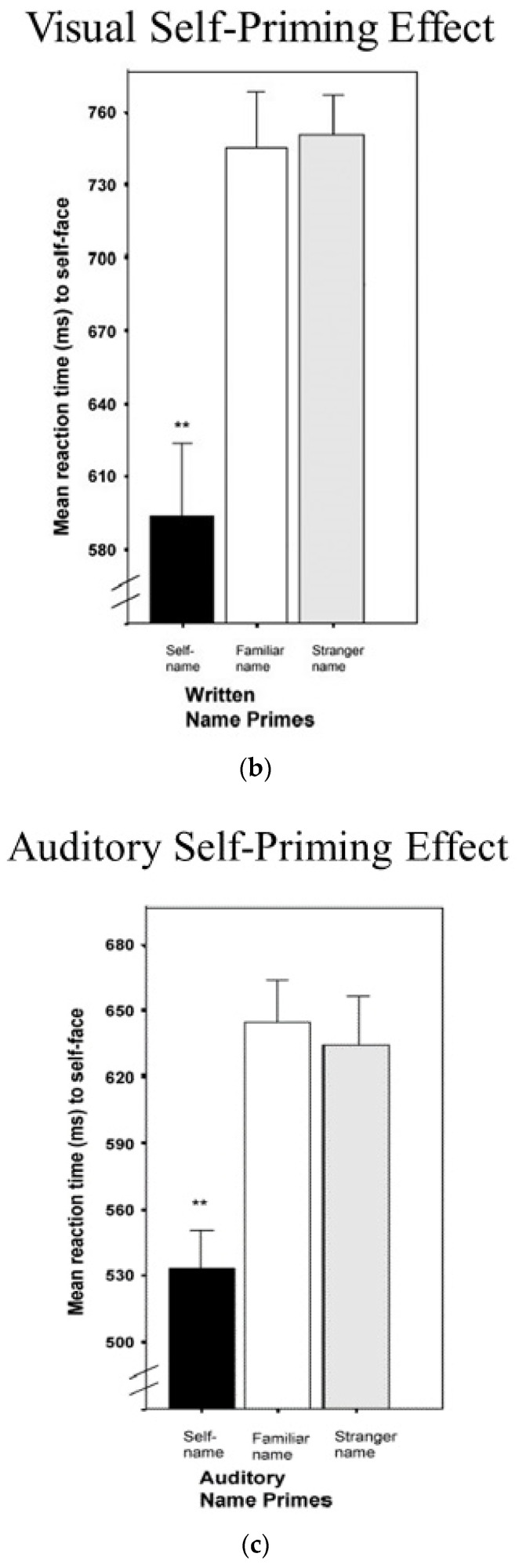
(**a**) Olfactory priming results; (**b**) Visual self-name priming; and (**c**) Auditory self-name priming (** *p* < 0.01).

A significant proportion of cognitive research using reaction time latencies involves assessing the effect of different primes, where a stimulus is briefly presented prior to a reaction time test to determine if the prime facilitates or interferes with processing. Platek, Thompson, and Gallup [2] investigated how information about the self affects reaction time latency to the self across different sensory and cognitive domains. First, they collected underarm body odor samples from participants and presented them along with a number of control odors, including other people’s body odors using an olfactometer, while participants were asked to identify visual images of themselves or others. Compared to control odors, priming one’s own smell with one’s own face sped up left-hand reaction times (see Figure 4). In the second experiment, participants were also asked to respond to self- or other faces after being primed by seeing either their own name, a familiar name, or a strange name. When participants were primed with their own name, reaction times to their own face became faster. In a final experiment, participants were primed by hearing their own name, a familiar name, or a stranger’s name prior to responding to self- or other faces. Again, it was the case that being primed in the auditory modality by hearing one’s own name increased the speed at which participants identified a face as being their own. 

These results have profound implications for how we think about the self. Take several common arguments as a case in point. In an attempt to explain the failure to find mirror self-recognition in some species, there are authors [39,40] who have reasoned that it may be possible to be self-aware in some modalities but not others. Conversely, if an organism can recognize itself in more than one modality, does that mean there are different self-concepts in different sensory domains (a visual sense of self, an olfactory sense of self, etc.)? The fact that processing information about the self in one sensory modality is affected by the presentation of information about the self in other modalities is compelling evidence for an underlying, integrated, cross-modal self-processing system. Therefore, it makes no sense to argue that there are separate, non-overlapping concepts of self in different modalities. Short of particular sensory deficits, it makes no sense to think that some organisms may be self-aware in some modalities but not others. After all, if you close your eyes, cover your ears, or hold your nose, your sense of self does not fade, fundamentally change, or disappear. Thus, the sense of self appears to integrate information about the self to achieve intermodal equivalence across different sensory domains.

Some investigators, such as Swartz [41] and Morin [42], contend that mirror self-recognition is not evidence for self-awareness, while Mitchell [43] and Povinelli [44] argue that apparent instances of self-recognition involve little more than matching motor cues to mirror feedback. The results of the present study stand in stark contrast to such claims. Procedures designed to prime the self in the visual, auditory, and olfactory modality all have one thing in common: they each were independently shown to facilitate self-face identification. As a consequence, self-recognition appears to be the byproduct of an underlying, multi-modal self-processing system that functions as a common denominator to all of these effects.

These results also imply that a sense of self does not emerge as a consequence of mirror self-recognition. Rather, a mirror simply represents a means of mapping what self-aware creatures already know about themselves and provides them with a new and novel means of seeing themselves as they are seen by others. 

Additionally, Li and Tottenham [45] showed that priming participants with an image of their own face facilitated recognition of emotions in other faces. They suggest that this finding links the ability to recognize emotions in others to a representation of our own face and emotional experience. 

While no cross-modal investigations of self-processing have been conducted in patients with schizophrenia, their families, or people that vary along the schizotypal personality spectrum, it would be interesting to see if patients with schizophrenia or individuals who score highly on the SPQ show an altered cross-modal response. One possible outcome could be that the deficiencies in self-processing would be magnified in these individuals, thus further increasing the difference in self-face latencies between groups. On the other hand, it is possible that these individuals may possess certain visual or particular face recognition deficiencies, and as such may show a compensatory increase in self-processing when confronted with other forms of self-related information. Given the increasing evidence of self and mental state attribution deficits along the schizophrenia spectrum, the latter assumption seems unlikely, but worthy of future research. 

Schizophrenics often see their own reflections in mirrors as independently alive, alien, or sinister [46]. They also have been observed talking to and laughing at their mirrored reflection as if it were another person [47]. In a now classic series of studies, Traub and Orbach [48,49] showed that schizophrenics had difficulty with a task that involved self-referent mirror use. The task involved rectifying a distorted mirror image of the subject, or an inanimate object using remote motorized controls attached to a metal mirror. Although schizophrenics were as good as the controls at adjusting the modified image of the inanimate object (a door) to eliminate the distortion, unlike control subjects they were unable to adjust the mirror to achieve an undistorted reflection of themselves. This suggests that rather than involving a deficit in mirror understanding, schizophrenia involves a deficit in self-processing. 

Additionally, Frith and his colleagues [7,50] have shown that schizophrenics occasionally report seeing nothing in their mirrored reflection (negative autoscopy) and are also unable to realize when instances of their own behaviors, such as speech, are self-initiated. Frith [50] has conceptualized schizophrenia as a disorder of mental states. He suggests that certain psychotic symptoms associated with schizophrenia may impair the ability to reason about other people’s mental states. This inability can then lead to social withdrawal, inappropriate social behaviors, and affective blunting. The literature has been fairly consistent in showing that schizophrenics perform worse on theory of mind tasks than non-psychiatric and psychiatric controls, as well as patients in remission [51]. For instance, Corcoran, Mercer and Frith [52] found that patients with schizophrenia perform poorer on a simple social inference task than normal and non-psychotic psychiatric control groups. Brunet, Sarfati, Hardy-Bayle, and Decety [53] used PET to investigate brain activation during a nonverbal theory of mind task. While control subjects showed significant cerebral activation in the right prefrontal cortex, activations of the right prefrontal cortex were not found in the schizophrenia group.

Platek and colleagues investigated the hypothesis that self-processing and mental state attribution are part of a shared behavioral and neurocognitive network that is impaired in schizophrenia-spectrum disorders [26]. They found that unaffected first-degree relatives of patients with schizophrenia took longer than controls to recognize their own face, but were more accurate in making self vs. other judgments. This was related to the level of schizotypy shown by the family members. Additionally, patients were more likely to misattribute the self to unfamiliar faces; i.e., when they made errors at classifying a novel face, they were more likely to indicate it was a self-face.

Patients with damage to the frontal cortex are not only impaired in the ability to recognize their own faces, but they show corollary deficits in self-evaluation and autobiographical memory [54]. Additionally, when patients have the anterior portion of the temporal lobe removed in order to treat intractable seizures associated with epilepsy, this impairs the ability to recognize their own face (slower reaction time and more errors) compared to personally familiar and famous faces (Platek, unpublished data). This finding, while inconsistent with a simple right-hemispheric model for self-processing, supports fMRI data [55] showing a larger, distributed network for self-processing that involves the anterior middle temporal gyrus (Platek, Scheiser, Glosser, Schneider, Irani, and Panyavin, unpublished data). Thus, the brain disorder data support the idea that self-recognition is impaired by deficiencies in frontal lobe processing that may be localized to specific regions (e.g., superior or inferior frontal gyri, as well as anterior temporal regions). 

## 6. Mindless Conversations

Most people have had experience with mindless conversations. It occurs when someone approaches you out of the clear blue and accidentally begins a conversation as if you were privileged to what they had on their mind. For example, one may have the experience of someone beginning a conversation such as “What do you think we ought to do about that?”, or “Gee, that makes me mad!” One’s likely response would be “Do about what?” or “What makes you mad?” It is a temporary failure on the part of the person that initiates the conversation to take into account the listener’s perspective. 

In contrast, schizophrenics often lose the capacity to take into account what other people know or intend to do, and as a consequence routinely begin conversations as if other people knew what was on their mind, and are perplexed and confused by the failure of other people to know what they are talking about.

## 7. The Absence of Mind

To illustrate what it would be like not to have a mind in the first place, imagine that you have a pet dog, and your dog returns from the woods following an encounter with a porcupine where its nose and its face are filled with porcupine quills.

With a concern for your dog’s well-being, you have no choice but to take steps to remove those barbed quills. Basically, you have two options. You could take your dog to a veterinarian to have the quills removed, or you could obtain a pair of pliers and attempt to remove the quills yourself. If you were to opt for the latter, it would prove to be an excruciating ordeal. It is not that you would experience any physical pain as a consequence, but given your prior experiences with pain, it would prove impossible not to empathize with what you assume to be going on inside the dog’s head as you rip out those quills and witness the dog’s reaction. It is not that you have to have prior experience with being quilled by a porcupine, but rather you could use your other prior experiences with pain to infer the pain being experienced by your dog.

The question this poses, however, is how would another unrelated dog react as it watches you remove those quills from your dog’s nose and face? Any veterinarian can tell you that dogs are oblivious to pain and suffering in other unrelated dogs. Therefore, it is likely the case that dogs can experience pain in pretty much the same way as you or I. What separates dogs from humans is that they are incapable of using their experience with pain to represent and infer painful experiences in other dogs, let along other creatures.

## 8. Testable Predictions and Future Directions

First, more research on patients with schizophrenia and self-processing is needed to clarify if all patients with schizophrenia show deficits in self-face processing and mental state attribution, or if these deficiencies are only seen in certain patient subtypes. Family studies would help elucidate the genetics of such deficiencies, as would direct genetic research. Services such as Ancestry or 23&Me could be used to do research on these topics if there was a way to develop an accurate web-based reaction-time recorder. 

Second, female patients with schizophrenia and those that score highly on the SPQ could be tested for olfactory self-recognition. We would predict that schizophrenic patients and women that score highly on the SPQ would be uniquely deficient at recognizing their own body odor. In contrast there ought to be no such differences in men, since men seem incapable of identifying their own body odor. 

Third, as we have outlined here, the sense of self is larger than just seeing yourself in the mirror, because it encompasses seeing your own face and name, hearing your own name, seeing adjectives that describe you, and so on and so forth., Therefore, an examination of multimodal self-processing could expand our knowledge of self-processing deficits, not only in patients with schizophrenia, but also in patients with autism and those who have had a frontal lobe injury or who have dementia. 

It would be interesting to see if, like dogs, patients with schizophrenia would remain unaffected by watching someone else experience an emotional or painful event. Cheng et al. [56] have shown that people use the same part of the brain when enduring pain that they experience themselves (with acupuncture needles) and when seeing pain experienced by another person [57]. We would predict that patients with schizophrenia, particularly those that demonstrated low scores on self-processing, would show reduced empathic pain awareness. 

The final issue is whether the self-face reaction time paradigm could be adapted for use with chimpanzees that pass the traditional mirror self-recognition test. Using something akin to an operant, variable time schedule of reinforcement, it ought to be possible, using a series of successive approximations, to encourage chimpanzees to press particular keys on a keyboard that correspond to their own face, familiar faces, or stranger faces as fast as possible. Initially the receipt of food rewards, such as raisins or peanuts, could be programmed to occur within 250 milliseconds of the appearance of a face on the computer monitor. Once performance was stable, you could then apply reinforcement through a series of successive approximations for responding more quickly to faces on the screen until you approximate human response latencies. Having achieved that, one of many next steps would be to restrict the option to press keys with either the right or the left hand to see if there is a lateralized reaction time latency advantage.

## 9. Conclusions

The use of self-face reaction time latencies is an important but often overlooked paradigm for assessing self-awareness that can be used as a more quantitatively sophisticated alternative to using a simple pass/fail mirror mark test. As we have shown in this paper, individual differences in self-face identification latencies can be used to identify a continuum of important, as well as dysfunctional individual differences in self-awareness and self-processing, and they provide a useful means of inferring underlying brain mechanisms. We are confident that self-face identification latencies can also be used to examine heretofore untapped dimensions of self-awareness in the future.

## Figures and Tables

**Table 1:** Traits used in self-trait study.

Self-Trait Identification	
Sincere	Considerate
Dishonest	Reliable
Understanding	Mature
Trustworthy	Lazy
Intelligent	Friendly
Dependable	Shy
Thougthful	Ambitious

**Table 2:** Effects of SPQ on self-face reaction time, faux pas theory of mind stories, and susceptibility to contagious yawning.

	Contagious Yawning	
	YES	NO
Self-face RT	771.4 ms	837.9 ms
(*p* < 0.01)		
*Faux Pas* TOM	97%	66%
(*p* < 0.05)		
SPQ score	15.33	36.00
(*p* < 0.05)		
r = −0.602, *p* < 0.01		

**Table 3:** Similarities between patients with frontal lobe damage and schizophrenia.

Symptoms	Brain Damage to Frontal Cortex	Schizophrenia
Impaired self-processing	Yes [30]	Yes [30]
Mental state attribution deficits	Yes [31]	Yes [32]
Autobiographical memory deficits	Yes [33]	Yes [34]
Wisconsin card-sorting test impairment	Yes [35]	Yes [36]
Olfactory deficits	Yes [37]	Yes [38]

## Data Availability

Not applicable.
